# Transcytosis-Driven Treatment of Neurodegenerative Disorders by mRNA-Expressed Antibody–Transferrin Conjugates

**DOI:** 10.3390/biomedicines12040851

**Published:** 2024-04-12

**Authors:** Sarfaraz K. Niazi, Matthias Magoola

**Affiliations:** 1 College of Pharmacy, University of Illinois, Chicago, IL 60612, USA; 2 DEI Biopharma, Kampala P.O. Box 35854, Uganda; dei@deiinternational.com

**Keywords:** mRNA, antibodies, nanobodies, neurological diseases, protein aggregates, protein misfolding, aducanumab, lecanemab, donanemab, cinpanemab

## Abstract

The recent setbacks in the withdrawal and approval delays of antibody treatments of neurodegenerative disorders (NDs), attributed to their poor entry across the blood–brain barrier (BBB), emphasize the need to bring novel approaches to enhance the entry across the BBB. One such approach is conjugating the antibodies that bind brain proteins responsible for NDs with the transferrin molecule. This glycoprotein transports iron into cells, connecting with the transferrin receptors (TfRs), piggybacking an antibody–transferrin complex that can subsequently release the antibody in the brain or stay connected while letting the antibody bind. This process increases the concentration of antibodies in the brain, enhancing therapeutic efficacy with targeted delivery and minimum systemic side effects. Currently, this approach is experimented with using drug-transferring conjugates assembled in vitro. Still, a more efficient and safer alternative is to express the conjugate using mRNA technology, as detailed in this paper. This approach will expedite safer discoveries that can be made available at a much lower cost than the recombinant process with in vitro conjugation. Most importantly, the recommendations made in this paper may save the antibodies against the NDs that seem to be failing despite their regulatory approvals.

## 1. Introduction

Neurodegenerative diseases (NDs) are complex disorders with multifactorial pathology that result in progressive damage to neuronal cells and loss of neuronal connectivity, ultimately leading to impaired mobility and/or cognition. Protein aggregation due to misfolding and oligomerization gives rise to extracellular or intracellular inclusions, a common hallmark for many NDs. Further spreading of these amyloid aggregates in the nervous system, like prion-based infections, hence, a prion-like mechanism, is often considered a significant element in the etiology of NDs [1]. In the past few decades, many of the genetic and biochemical causes underlying NDs associated with protein aggregation were uncovered, leading to the distinction between rarer familial forms, where disease-causing mutations are genetically inherited, and the more common sporadic forms, where genetic and environmental risk factors drive the pathogenesis [2]. In both cases, the affected proteins are found enriched in pathological aggregates, highlighting their importance in the manifestation of the disease. However, despite the accumulated knowledge and the many clinical trials in which attempts were made to alleviate protein aggregation, no therapeutic strategy has been broadly accepted to cure any of the NDs. This led many scientists to question whether protein aggregation was central to ND etiology or a manifestation of other underlying causes [3,4]. Nonetheless, collectively, the work of the past decades generated a more complex understanding of how each aggregation-prone protein engaged with many critical cellular pathways. This review aims to provide an overview of these intricate connections by combining core findings and more recent discoveries.

For each ND, different sets of genes are typically found mutated in the familial forms, and different brain regions and cell types are initially affected. For example, Huntington’s Disease (HD) and spinocerebellar ataxia type 1 (SCA1) are linked to the expansion of the CAG repeat of the huntingtin (HTT) and ataxin 1 (ATXN1) genes, respectively, resulting in proteins with an unusually long polyglutamine (polyQ) tract that is very prone to aggregation and causes intracellular deposits in striatal neurons [5,6]. In Alzheimer’s disease (AD), two different types of deposits are observed. The aberrant cleavage products of the transmembrane protein amyloid-β (Aβ) precursor protein (APP) form extracellular plaque deposits in the temporal and parietal brain regions. In contrast, the protein tau accumulates intracellularly in neurofibrillary tangles [7]. In Parkinson’s disease (PD), the primarily affected brain area is the substantia nigra (SN), where α-synuclein aggregates are found to accumulate in dopaminergic neurons [8]. In ALS, cellular aggregates of superoxide dismutase 1 (SOD1), RNA-binding protein FUS (FUsed in Sarcoma), and TAR-DNA-binding protein 43 (TDP-43) have been identified in motor neurons of the primary motor cortex, brainstem, and spinal cord [9].

Protein misfolding and aggregation of disease-associated proteins are facilitated by mutations and post-translation modifications (e.g., phosphorylation and protein cleavage) that avert the formation of the native protein structure. At the same time, in some other cases, misfolding can also seemingly occur sporadically without a clear explanation. Aggregation is first typically initiated by a seed or/and an oligomer, in which sequence-specific elements of the misfolded protein interact to adopt a non-native conformation, which can then convert other proteins into the toxic form. In many cases, the oligomerization of misfolded proteins leads to the formation of amyloid fibrils with a distinctive β-sheet structure that arises when soluble oligomers assemble into small protofibrils [10]. When more proteins are converted into non-native forms, these protofibrils become longer fibrils that can then form more extensive cellular inclusions visible by light microscopy. Recently, it has been proposed that oligomerization may be favored by liquid–liquid phase separation of aggregation-prone proteins [11]. Moreover, it is evident that there are different polymorphs for most amyloid fibrils in vitro and in vivo (polymorph is a term used to indicate the capacity of a polypeptide to generate fibrils with different structures) [12,13].

The protein fibrils formed would be expected to be removed by the autoimmune responses. Still, the less efficient production of antibodies in the brain must be supported by systemic entry of the BBB [14,15], leading to extensive research and development of these therapies.

An emerging therapeutic strategy in neurodegenerative diseases involves designing antibodies to target and clear abnormal protein aggregates. Two such monoclonal antibodies (mAbs), aducanumab and lecanemab, have received accelerated approval from the US FDA for the treatment of AD [16,17]; however, aducanumab is discontinued effective 2024, without reasons and with the claim that it is not due to issues with safety or efficacy [18]. Another monoclonal antibody, donanemab, was in the advanced stages of development for patients with early AD [19]. Still, the FDA has delayed its approval, likely due to a lack of proof of efficacy [20]. Another drug, Relyvrio (Cinpanemab), for ALS, is being recalled due to lack of efficacy [21,22]. The data reporting on clinicaltrials.gov show that around 250 interventional studies treat NDs involving antibodies [23]. However, the status of the failure of antibodies to provide a treatment solution for NDs requires rethinking whether an antibody alone can be effective in NDs without adding functions to promote their entry across the BBB.

The options available to enhance the entry of antibodies into the brain include invasive techniques, including intra-cerebral injection, convection-enhanced delivery, and intra-cerebroventricular infusion [24]. The BBB can be disrupted using bradykinin analogs, ultrasonography, and osmotic pressure [25]. Adding microbubbles makes these techniques more effective [26,27]. Pharmacological techniques involve encapsulating medications into liposomes or chemically modifying pharmaceuticals to lipophilic molecules [28]. Opsonization and drug delivery by nanoparticles across the blood–brain barrier is the process in which the drug is adsorbed onto the particles passively [29]. Intranasal delivery routes can bypass the BBB, offering a direct path to the CNS [30]. While all these approaches can be effective, none allow for a consistent and predictable response, which is one reason why these approaches have not been used in developing antibodies against NDs.

## 2. Transcytosis

**Figure 1:**
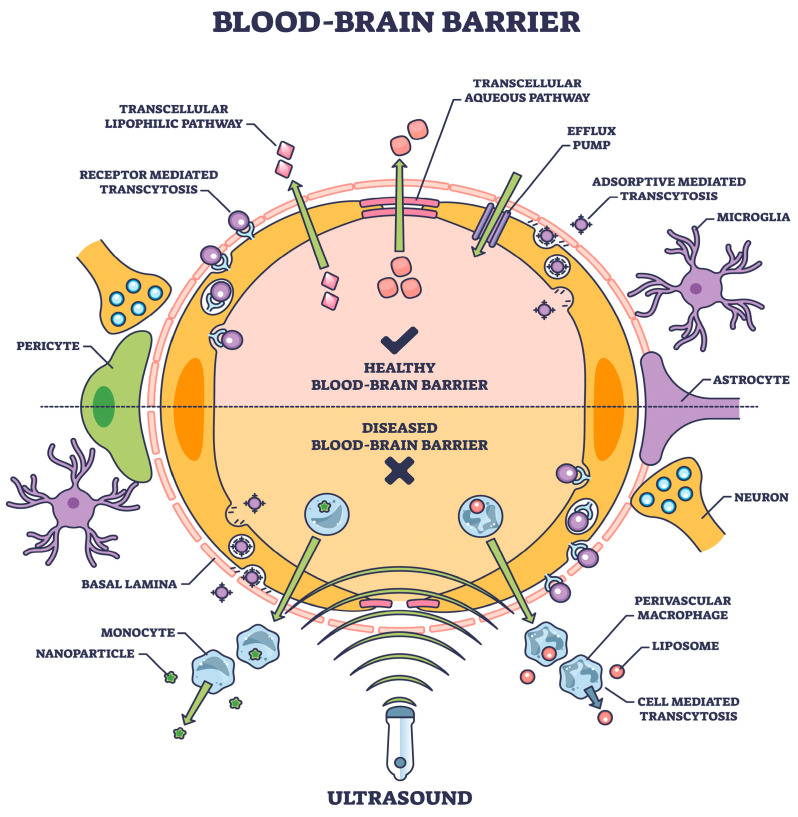
The blood–brain barrier means expediting natural or induced transport as a therapeutic measure [shutterstock_2304653921 Licensed].

One approach, a physiological procedure of transporter-mediated delivery, receptor-mediated transcytosis, and adsorptive-mediated transcytosis [31], offers a viable choice if the transcytosis mechanism is part of the drug structure. This is one of the several mechanisms involved in transporting various chemicals into the brain (Figure 1), as described in detail elsewhere [32].

The BBB is a highly selective semipermeable border that separates the circulating blood from the brain and extracellular fluid in the CNS. The BBB is composed of microvascular endothelial cells, which tightly regulate the movement of molecules, ions, and cells between the blood and the brain, unlike in other body parts. This regulation is crucial for maintaining the brain’s stable environment, which is necessary for proper neuronal function. The BBB performs several critical roles, including protecting the brain from harmful substances in the blood, regulating the transport of essential nutrients, and maintaining a constant environment for the brain [33,34].

Substances allowed to cross the BBB include water, some gases (like oxygen and carbon dioxide), and lipid-soluble substances (e.g., alcohol, nicotine, and caffeine) that can diffuse through the cell membranes of the endothelial cells. Additionally, specific transport mechanisms exist for essential nutrients that the brain requires but are not lipid-soluble, such as glucose and amino acids. These substances use facilitated diffusion and active transport mechanisms to cross the BBB. Conversely, most large molecules, including many drugs and pathogens, are generally prohibited from passing through the BBB. This selective permeability presents a significant challenge for drug delivery to the brain, particularly for the treatment of brain diseases such as Alzheimer’s and Parkinson’s [35].

However, the BBB can be compromised under certain pathological conditions, such as inflammation, ischemic stroke, or high blood pressure, allowing for the substances usually blocked to enter the brain. This breach in the BBB’s integrity can contribute to the progression of various neurological disorders by permitting toxins, pathogens, and immune cells to invade and damage brain tissues [36].

Misfolded proteins in AD and PD follow a well-defined connectomics-based spatial progression. Several anti-tau and anti-alpha synuclein (aSyn) antibodies have failed to provide clinical benefit in clinical trials despite substantial target engagement in the experimentally accessible cerebrospinal fluid (CSF). The proposed mechanism of action is reducing neuronal uptake of oligomeric protein from the synaptic cleft. Integration with a physiologically based pharmacokinetic (PBPK) model has allowed for the simulation of clinical trials of anti-tau antibodies gosuranemab, tilavonemab, semorinemab, and anti-aSyn antibodies cinpanemab and prasineuzumab [37].

Some antibody fragments have many advantages over monoclonal antibodies, such as small sizes, lack of the crystallizable fraction (Fc), etc. There are three main antibody fragments: single-chain variable fragments (scFvs); Fab fragments; and single-domain antibody fragments. Nanoparticles can facilitate the entry of drug molecules across the blood–brain barrier, making them excellent carriers. Various kinds of nanoparticles have been applied in the treatment of AD. The combination of nanoparticles and antibody fragments against amyloid-β can be used to diagnose and treat Alzheimer’s disease, including antibody fragments against amyloid-β in AD [38].

Understanding the mechanisms of BBB permeability and its role in health and disease is a critical area of research, with implications for developing new therapeutic strategies for various neurological conditions.

Receptor-mediated transcytosis (RMT) is a highly specialized process that allows for the transport of specific molecules, particularly antibodies, across the BBB via receptors on the surface of endothelial cells. This process is vital for the delivery of essential nutrients and biomolecules that are not lipid-soluble and cannot diffuse via the endothelial cell membranes of the BBB. Several well-documented examples of RMT into the brain involve the transport of transferrin, insulin, and low-density lipoprotein (LDL) related proteins.

### 2.1. Transferrin

**Figure 2:**
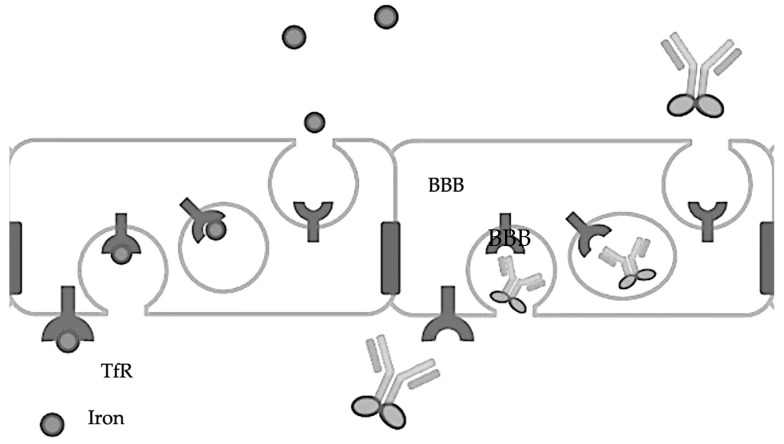
Transferrin-fused antibodies are transported across the BBB.

The transferrin receptor (TfR) is involved in the transport of iron into the brain via transferrin, a protein that binds iron tightly. The TfR-mediated transferrin transport across the BBB is crucial for maintaining iron homeostasis in the brain, as iron is essential for various brain functions, including oxygen transport, DNA synthesis, and electron transport [39,40]. The TfR [41] can effectively transport therapeutic levels of antibodies across the BBB [42] by attaching them to transferrin or transferrin-mimicking peptides, thereby facilitating their crossing of the BBB [43,44]. Another target is the insulin receptor (IR), which, like the TfR, can mediate the transport of antibodies across the BBB, offering a pathway for therapeutic intervention [45]. So, theoretically, a conjugate of insulin and an antibody should enhance the entry of antibodies across the BBB, though no such studies have been reported (Figure 2).

### 2.2. Insulin

The insulin receptor facilitates the transport of insulin across the BBB through RMT. Insulin in the brain is essential for multiple brain functions, including neuronal growth, survival, and regulating appetite and cognitive functions. Insulin transport into the brain is believed to play a role in the central regulation of peripheral glucose metabolism (Banks, 2004 [46]).

### 2.3. Low-Density Lipoprotein (LDL) Receptor-Related Proteins (LRP)

LRP1 and LRP2 (as megalin) transport various ligands across the BBB, including vitamin A-binding protein and apolipoprotein E-containing lipoproteins. These processes are essential for delivering vitamins, cholesterol, and other lipids crucial for brain development, maintenance, and function [47]. The low-density lipoprotein receptor-related protein-1 (LRP1) also serves as a conduit for the delivery of certain therapeutics into the brain, capitalizing on its role in transporting various molecules, including lipoproteins and amyloid-beta precursors [48]. LRP1, involved in transporting various endogenous ligands, including apolipoprotein E-containing lipoproteins, has been identified as an alternative pathway for BBB crossing. This receptor participates in the clearance of amyloid-beta from the brain and has been implicated in the transport of other therapeutic agents [49].

The other two proteins, GLUT1 and P-glycoprotein (P-gp), have not been well studied or found effective [35].

These examples of RMT highlight the complexity and specificity of the mechanisms that regulate the transport of molecules across the BBB. Understanding these pathways is crucial for developing strategies to enhance drug delivery to the brain, particularly for treating neurological diseases.

The decision to use transferrin or LRP1 as a delivery mechanism would, thus, be based on the specific requirements of the therapeutic agent, including its size, required dosage, and targeted area within the brain [50].

## 3. Transcytosis Approach

Using transferrin for the targeted delivery of therapeutic agents, including antibodies, to treat neurodegenerative disorders is based on exploiting the TfR pathway. This pathway facilitates crossing the BBB through receptor-mediated transcytosis. While numerous research efforts have been focused on utilizing this pathway to treat neurodegenerative diseases, such treatments’ development and clinical application are still in the early stages. AD research has explored using transferrin-conjugated nanoparticles or therapeutic agents to enhance delivery across the BBB. These approaches target amyloid-beta (Aβ) plaques or tau proteins, characteristic of AD pathology. For instance, a study by Liao et al. [51] investigated the use of transferrin-conjugated nanoparticles to deliver siRNA specifically targeting BACE1, a critical enzyme in producing Aβ, demonstrating successful delivery and therapeutic effects in a mouse model of AD. For PD, where dopaminergic neurons are progressively lost, research has focused on delivering neuroprotective agents directly to the affected brain regions. While direct examples of transferrin-antibody conjugates for PD are limited, the concept has been considered for delivering neurotrophic factors that could potentially halt or reverse neuronal degeneration.

Biologic drugs are large molecules that do not cross the BBB. Brain penetration is possible following re-engineering the biological drug as an IgG fusion protein. The IgG domain is a mAb against an endogenous BBB receptor such as the TfR. The TfRmAb acts as a molecular Trojan horse to ferry the fused biological drug into the brain via receptor-mediated transport on the endogenous BBB TfR. The BBB delivery of biologic drugs is possible following re-engineering as a fusion protein with a molecular Trojan horse such as a TfRmAb. The efficacy of this technology will be determined by the outcome of future clinical trials [52].

Transferrin conjugates have shown promise in the treatment of neurodegenerative disorders, demonstrating the effectiveness of transferrin as a delivery vector for nerve growth factor (NGF) in targeting the central nervous system (CNS) and improving recognition and memory in neurodegenerative diseases [53,54].

Transferrin receptor antibody-NGF conjugate prevented the degeneration of cholinergic striatal neurons in a model of Huntington’s disease [55]. The ability of transferrin to transport neurotrophic factors across the blood–brain barrier showed that an OX-26-GDNF conjugate enhanced the survival of spinal cord motor neurons [56]. However, the potential role of transferrin in the uptake of neurotoxic agents remains a concern [57].

A recent study shows that mucopolysaccharidosis type I causes systemic accumulation of glycosaminoglycans due to a genetic deficiency of alpha-L-iduronidase, which results in progressive systemic symptoms affecting multiple organs, including the central nervous system (CNS). A genetically modified protein consisting of IDUA fused with humanized anti-human TfR antibody (lepunafusp alfa) shows distribution into the brain [58], bringing about systemic reductions in heparan sulfate and dermatan sulfate concentrations.

Single-domain shark antibodies that bind to the TfR1 on brain endothelial cells have been used to shuttle antibodies and other cargos across the BBB to the brain. The TXB4 brain shuttle was fused with a TrkB neurotrophin receptor agonist antibody for these studies. The TXB4-TrkB fusion retained potent agonist activity at its cognate receptor and, after systemic administration, showed a 12-fold increase in brain levels over the unmodified antibody [59].

Pabinafusp alfa is a novel enzyme drug that crosses the blood–brain barrier by transcytosis via transferrin receptors [60].

Erythropoietin, a hematopoietic growth factor and a promising therapy for Alzheimer’s disease, has low permeability across the blood–brain barrier. The transferrin receptor antibody fused to erythropoietin, a chimeric monoclonal antibody that ferries EPO into the brain via the transvascular route [61].

The TfR has remained a popular target since its original description for this purpose, although the clinical progression of TfR-targeted drug constructs or nanomedicines remains unsuccessful [62].

One issue related to using TfR-targeting in nanomedicines is the efficient tuning of the ligand density on the nanoparticle surface [63].

Targeting TfR on the surface of brain capillaries has been a popular strategy to give a preferential accumulation of drugs or nanomedicines, but several aspects of this targeting strategy remain elusive; monovalent ligands may be beneficial for obtaining transcytosis of TfR-targeted nanomedicines across the BBB, which is relevant for future design of nanomedicines for brain drug delivery [64].

Recombinantly fused two single-chain variable fragments (scFv) of the TfR antibody 8D3 to the light chains of mAb158, an antibody selectively binding to Abeta protofibrils, which are involved in the pathogenesis of AD, markedly increasing mAb158 brain uptake, which makes it a strong candidate for improved Abeta immunotherapy and as a PET radioligand for early diagnosis and evaluation of treatment effect in AD [65].

Monoclonal antibodies directed against the TfR have been proposed as potential carrier candidates [66]. Anti-amyloid antibodies (AAA) are under development as new therapeutics that disaggregate the amyloid plaque in the brain in AD. An AAA was re-engineered for receptor-mediated transport across the BBB via the endogenous BBB TfR [67].

## 4. Conjugation

This antibody–transferrin conjugation strategy is particularly valuable in the development of targeted drug delivery systems as it can potentially enhance the ability of therapeutic agents to cross the blood–brain barrier via receptor-mediated transcytosis, exploiting transferrin’s natural ability to bind to transferrin receptors that are abundantly expressed on the surface of brain capillary endothelial cells [68].

Connecting an antibody to a transferrin protein requires meticulous bioconjugation techniques. This process starts with the purification and accurate quantification of both proteins to ensure the success of subsequent reactions [69]. The transferrin protein undergoes chemical activation to introduce functional groups capable of forming stable bonds with the antibody. This step typically employs cross-linking agents. There are several ways to create an antibody–transferrin conjugate; the most common method is to bind an antibody to transferrin in vitro; this process involves a series of biochemical techniques aimed at creating a conjugate that can be used for various research and therapeutic purposes. This process, known as antibody–transferrin conjugation, typically starts with the purification of the antibody and transferrin to ensure their compatibility and functionality in subsequent steps. One common method for conjugation uses bifunctional cross-linking agents, such as N-succinimidyl 3-(2-pyridyldithio)propionate or succinimidyl 4-(N-maleimidomethyl)cyclohexane-1-carboxylate, which can form stable bonds with amine groups on the antibody and thiol groups on the transferrin [70]. However, creating a flexible link to remove constraints is desirable so that both molecules can interact independently. These linkers can include polyethylene glycol spacers, which not only increase the flexibility of the conjugate but also enhance its solubility and reduce immunogenicity. The flexibility provided by such linkers can be crucial for allowing the proteins to move and interact with their targets effectively [71].

The conditions under which transferrin is activated—temperature, pH, and reaction time—are carefully controlled to preserve protein integrity and function. The antibody is then introduced to the activated transferrin under conditions favoring the coupling reaction between the functional groups of both proteins. Optimal conditions are maintained to facilitate effective conjugation while preserving the biological functions of each molecule. Post-reaction, the conjugation mixture necessitates purification to isolate the desired antibody–transferrin conjugate from unreacted components and by-products. Techniques such as dialysis, gel filtration chromatography, or affinity chromatography are commonly employed for this purpose, each selected based on the specific properties of the conjugate and the reagents used. Finally, the success and efficiency of the conjugation process are established using analytical techniques like SDS-PAGE, Western blotting, or mass spectrometry to verify the formation of the conjugate and assess its molecular weight and purity. The biological activity of the conjugate, particularly its target specificity and cell-binding efficiency, is then tested in relevant bioassays to ensure that the functionalities of both the antibody and transferrin have been retained post-conjugation. This meticulous validation ensures that the final product is suitable for its intended diagnostic or therapeutic applications [72].

## 5. Linkers

Several considerations must be considered when selecting a linker to bind an antibody to transferrin to facilitate entry into the brain. First and foremost, it is crucial to understand the mechanisms by which molecules, including antibodies, can cross the BBB [73]. TfRs are one of the targets for transporting molecules across the BBB. Therefore, conjugating antibodies to transferrin can facilitate brain entry via receptor-mediated transcytosis. Secondly, the linker should be designed to be stable during circulation but cleavable in the brain milieu to release the antibody from transferrin. Various cleavable linkers, such as protease-sensitive or pH-sensitive linkers, can be considered [52,74]. Thirdly, the linker should maintain stability in the bloodstream to prevent premature release of the antibody–transferrin conjugate. Stability can be influenced by factors such as serum proteases and pH [75].

Additionally, the linker should be designed to be cleavable within the unique microenvironment of the BBB, which may have different enzymatic activities or pH compared to other tissues [76]. Once a linker design is proposed, it is crucial to validate its efficacy in facilitating brain entry and cleavage in preclinical models [77]. Optimization may be necessary to achieve the desired pharmacokinetics and brain distribution. Finally, assessing the safety profile of the linker and ensuring the specificity of brain targeting are important considerations to minimize off-target effects and potential toxicity [73].

The pH-sensitive linkers play a crucial role in drug delivery systems, as they are designed to remain stable at physiological pH but become cleavable under acidic conditions typically found in endosomes or lysosomes. One example is hydrazone linkers, which form via the reaction between a hydrazide and a carbonyl group under acidic conditions. They exhibit stability at neutral pH but undergo hydrolysis in acidic environments, facilitating linker cleavage. This mechanism has been utilized in various drug delivery systems, including liposomes and polymer conjugates [78]. Another commonly used pH-sensitive linker is the acetal linker, which remains stable at neutral pH but undergoes acid-catalyzed hydrolysis to release the payload under acidic conditions. This linker has found applications in polymeric micelles and nanoparticles, offering controlled drug release in response to pH changes [79]. Vinyl ether linkers represent another type of pH-sensitive linker, remaining stable at neutral pH but rapidly hydrolyzing under acidic conditions. They have been employed in antibody-drug conjugates and prodrugs for targeted drug delivery, showcasing their versatility in pH-responsive drug release systems [80].

Additionally, ortho ester linkers are stable at neutral pH but undergo rapid hydrolysis under acidic conditions. This property has been exploited in various drug delivery systems, including polymeric nanoparticles and liposomes, enabling controlled intracellular drug release in response to acidic environments [81]. These examples highlight the significance of pH-sensitive linkers in achieving controlled drug release at specific sites within the body, enhancing the efficacy and safety of drug delivery systems.

Designing peptide linkers for targeted cleavage in the brain involves intricate knowledge of protease activity and substrate specificity. Examples include neprilysin (NEP) and its substrates [82], tPA and plasmin system [83], peptide linker design in therapeutic proteins [84], and bioinformatics tools for linker design [85].

## 6. Molecular Modeling and Testing

A molecular docking exercise can help select a particular fragment. Molecular docking is a pivotal bioinformatics technique that predicts the preferred orientation of one molecule to a second when bound to each other to form a stable complex. Understanding the interaction between antibody fragments (such as Fab, F(ab’)2, or scFvs) and target proteins like amyloid-beta or alpha-synuclein is crucial. Tools like AutoDock Vina (https://vina.scripps.edu/ accessed on 1 March 2024), one of the most cited and utilized software in molecular docking studies, enable researchers to simulate the docking process and evaluate the binding affinity between molecules [86,87]. Another widely used platform is Schrödinger’s suite, which offers comprehensive tools, including Glide, for high-throughput virtual screening and precise docking [88]. For specifically dealing with proteins like amyloid-beta and alpha-synuclein, Rosetta’s protein–protein docking protocol has been effectively employed to predict the structure of protein complexes in a near-native state [89]. A more straightforward approach is to use HADDOCK [https://wenmr.science.uu.nl/haddock2.4/ accessed on 14 March 2024] and PRODIGY [https://bianca.science.uu.nl/prodigy/. accessed on 14 March 2024] platforms.

These tools collectively provide a robust suite for predicting and analyzing the molecular interactions between antibody fragments and their specific targets, offering insights into the mechanism of action and facilitating the optimization of therapeutic antibodies for neurodegenerative diseases. Through applying these bioinformatics tools, researchers can gain a deeper understanding of the complex interactions at play, guiding the development of more effective and targeted treatments.

To test whether a projected conjugate is effective, isotope labeling can be exploited as a powerful technique for tracing the entry and distribution of proteins and their fragments, such as therapeutic antibodies, into the brain. This method involves labeling proteins with stable or radioactive isotopes, such as ^13^C ^15^N, or radioisotopes like ^131^I, which can be detected using various imaging and analytical techniques. A prominent application of this approach is in studying the BBB permeability and the biodistribution of therapeutics targeting neurological conditions. One commonly used method is positron emission tomography (PET), where isotopically labeled proteins can be visualized in vivo, providing real-time data on their brain uptake. For instance, labeling antibody fragments targeting amyloid-beta or alpha-synuclein with ^11^C or ^18^F allows for the PET imaging of their distribution within the brain, offering valuable insights into their therapeutic potential and mechanism of action [90].

Additionally, stable isotope labeling with amino acids in cell culture is used in proteomics to incorporate isotopically labeled amino acids into proteins, enabling the quantitative analysis of protein dynamics and interactions through mass spectrometry. This approach can elucidate the trafficking and metabolism of therapeutic proteins and peptides within the brain tissue [91]. These isotopic labeling methods, combined with advanced imaging and analytical tools, provide a robust framework for understanding how therapeutic proteins and their fragments cross the BBB and interact with target sites in the brain, contributing significantly to developing effective treatments for neurodegenerative diseases.

## 7. mRNA-Based Transcytosis

This process involves the ribosome moving along the mRNA strand, reading its sequence three nucleotides (a codon) at a time. Each codon specifies a particular amino acid, the building block of proteins. Transfer RNA (trNA) molecules carrying specific amino acids match up with the codons on the mRNA strand via their anticodon region. As the ribosome facilitates this matching, it also catalyzes the formation of peptide bonds between the amino acids, elongating the protein chain. This continues until the ribosome encounters a stop codon, signaling the end of the protein-coding sequence. The newly formed protein then folds into its functional three-dimensional structure and begins performing its role in the cell. As suggested in this paper, the protein could be an antibody, a fragment of an antibody, or a conjugate with transferrin protein. This should be compared with the recombinant manufacturing of proteins and in vitro conjugation. Recombinant manufacturing involves complex cloning, transformation, and purification steps, requiring significant biotechnological infrastructure and expertise. The mRNA-based production simplifies some of these steps by directly utilizing the host’s cellular machinery for protein synthesis, bypassing the need for culturing cells and extracting the protein. The mRNA synthesis can be faster and more easily scaled than traditional recombinant protein production, which is advantageous for rapid response scenarios, such as vaccine development during a pandemic. While recombinant protein production is a well-established and versatile method suitable for a wide range of proteins, mRNA-based production is particularly effective for applications where direct expression within the host is desired, such as vaccine development or delivery of antibody–transferrin conjugates. Presently, antibodies to treat NDs are produced by a recombinant engineering process that is expensive and takes a long time to establish the safety and efficacy of an antibody. The bioconjugation process then follows their production and purification. However, the conjugate can be produced by in vivo translation using mRNA; in that case, the conjugation can be made using a variety of linkers, such as glycine–serine-rich linkers often used in fusion proteins to provide flexibility and distance between functional domains [92]. The (Gly-Gly-Gly-Ser)n linkers provide highly flexible links to minimize interference with the biological activity of the linked domains [93]. The elastin-like polypeptides provide elasticity and flexibility [94]. The helix-forming linkers adopt a helical structure to bridge protein domains to provide a balance between flexibility and stability [95]. Some chemical linkers do not apply to the mRNA translation of antibody–transferrin conjugate. Still, as shown below, the glycine–serine linker provides sufficient stability and does not interfere with the binding.

**Figure 3:**
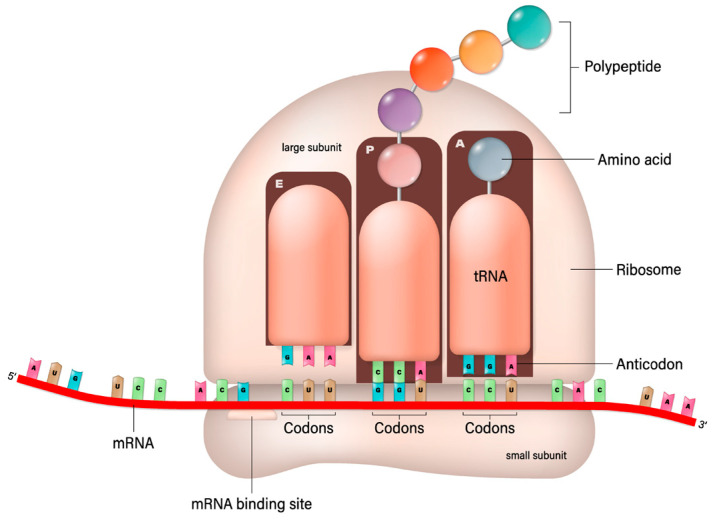
mRNA-based translation of proteins [Shutterstock license].

The mRNA technology is preferred for its faster and lower cost development and does not face the complexity of the upstream and downstream processes and post-translational modification consideration. The mRNA sequence can be synthesized by in vitro transcription in a cell-free environment: a linearized, plasmid DNA molecule is combined with ribonucleotides in the presence of bacteriophage RNA polymerase (of which T7 is the most widely used); the polymerases then recognize the promoter region in the DNA template and synthesize the RNA transcripts in the presence of ribonucleotides [96,97] (Figure 3).

### mRNA Design

The mRNA template used to deliver antibodies is shown in Table 1, wherein the antibody heavy and light chains are linked using the GGGS link, and the transferrin is connected. The final sequence of the mRNA is presented for the antibodies currently in use or waiting to enter the market. For mRNA translation, a single chain is structured as follows: Transferrin (PO2787)-GSGSGSGS-Heavy chain-linker-light chain, ending in a linear chain sequence; the linker need not break provided there is enough flexibility that can be tested using bioinformatics tools.

Interestingly, many of the currently approved or tested antibodies against NDs also show activity using single-chain variable fragments (scFvs), Fab fragments, and single-domain antibody fragments that provide a remarkable opportunity for further development of the current antibodies as these smaller sizes are more functional and more accessible to construct.

## 8. Regulatory

The ND products mentioned above that have failed or are failing under development to treat NDs have cost billions of dollars. Such losses will dampen the research in the field of ND treatment by antibodies; however, these products can be rejuvenated using a regulatory plan based on the FDA’s new guideline [98] that encourages innovative approaches that apply to the regulatory plan. While conjugates have been designed with transferrin in vitro, a much lower cost and faster development can be achieved using the mRNA technology. For example, suppose the current failing drugs are conjugated with transferrin and expressed through mRNA. In that case, these will still be new biological license applications, but with fewer and shorter studies that can be readily affordable and completed quickly.

To establish the proof of efficacy, as suggested above, a pharmacokinetic analysis based on a radioactive drug [99] in animal species should suffice; the process involves making the current product radioactive and conjugating it with transferrin first in vitro, comparing the radio image with the same molecule but without conjugation, before transforming the process to mRNA. Since these studies do not involve humans, they can be conducted without regulatory involvement within a very short time [100] and with almost negligible cost. Once the proof of concept is established, the regulatory agencies should be approached with a development plan that includes efficacy testing. However, these studies could be minimized by using a “generally accepted scientific knowledge-based” presentation based on comparable animal studies [98].

## 9. Challenges

The initiation of new mRNA-based protein production faces several significant challenges and difficulties spanning technical, regulatory, and intellectual property landscapes. First, the patent landscape for mRNA technology is complex and densely populated, with key intellectual property rights held by a few entities, which can hinder new entrants from accessing foundational technologies without navigating costly licensing agreements [101]. Secondly, the expertise required to design, optimize, and manufacture mRNA constructs and the lipid nanoparticles crucial for their delivery is highly specialized, representing a significant barrier to entry [102]. Moreover, rigorous testing and regulatory approval processes, which include preclinical studies, clinical trials, and manufacturing quality control, are time-consuming and resource-intensive [103]. These processes are critical to ensuring the safety and efficacy of mRNA-based therapies but represent a substantial upfront investment with no guaranteed outcome. Developing mRNA technology for new proteins also requires overcoming scientific challenges such as ensuring stability, efficient cellular uptake, and precise control over protein expression levels [104]. Each area presents challenges, making initiating new mRNA-based protein production complex and multifaceted.

The advancement and global dissemination of mRNA technology have been significantly supported by a network of international agencies and partnerships committed to public health innovation and equitable vaccine access. The Coalition for Epidemic Preparedness Innovations, launched in 2017, has emerged as a key player in funding mRNA vaccine research, emphasizing the importance of readiness for future epidemics [105].

Similarly, the World Health Organization has been central to coordinating the global health response, including efforts to ensure equitable distribution of mRNA vaccines via initiatives aimed at technology transfer to low- and middle-income countries (LMICs) [106].

GAVI, the Vaccine Alliance, through its COVAX facility, has worked tirelessly to facilitate fair access to COVID-19 vaccines, underscoring the role of global collaborations in addressing vaccine equity [107]. Non-profit organizations like PATH and philanthropic entities such as The Bill & Melinda Gates Foundation have also supported mRNA vaccine development and addressed the logistical and economic challenges of vaccine deployment in underserved regions. The WHO’s mRNA Technology Transfer Hub initiative also represents a concerted effort to enhance manufacturing capacity for mRNA vaccines globally, aiming to democratize production and ensure broader vaccine access [108]. These resources make the deployment of technology more accessible, yet the regulatory cost remains formidable, a primary concern for developing new biological drugs. However, this can be reduced by producing multiple products over a short period and testing them before entering phase I stages.

## 10. Conclusions

Therapeutic antibodies represent one of the fastest-growing segments in the pharmaceutical industry [109], expanding the scope to various antibody types, including nanobodies and Ab fragments, with optimized affinity, stability, and solubility [110].

NDs frequently involve disordered proteins that the inefficient immune system of the brain is not capable of removing, leading to scores of untreatable disorders. Much of current research is focused on designing antibodies, and a few have been approved, yet their use remains limited due to their poor entry into the brain, even as nanobodies. The effectiveness of these antibodies can be substantially higher if they are conjugated with transferrin protein as a choice modification to enhance their entry into the brain. Currently, the dose of antibodies entering the brain is less than 1%; thus, any change brought by improved transit across the brain will dramatically change their efficacy. In our opinion, this modification should be a standard approach for all future treatments since this provides a more reproducible means of promoting the entry of antibodies into the brain compared to dozens of other invasive and noninvasive techniques [32].

We recommend that aducanumab and lecanemab, which have received accelerated approval from the US FDA for the treatment of AD [16,17], donanemab [19], and Relyvrio (Cinpanemab) for ALS [21,22] can be good targets to investigate their transferrin conjugates expressed by mRNA as a logical option to rejuvenate their status and frontline ND treatment.

The advantages of mRNA over recombinant process are well established [111]; besides the safety of RNA products, as they do not enter the nucleus, the benefit of developing these products at a fraction of the cost of developing recombinant products and with the speed that had never been possible in any new drug development should be the choice approach to save these drugs. While safety and efficacy testing should never be comprised of the development cost, making these products accessible to the developing world is a dire humanitarian cause [112], particularly when treating NDs [113]. Since the development of mRNA is fast and costs much less than recombinant production [102], we have estimated that per dose cost of goods should not be higher than one dollar [102,111,114], not counting the amortization of the development cost, but that too is much lower than the billions of dollars spent on recombinant drugs.

## Figures and Tables

**Table 1:** mRNA template to produce antibody–transferrin conjugates.

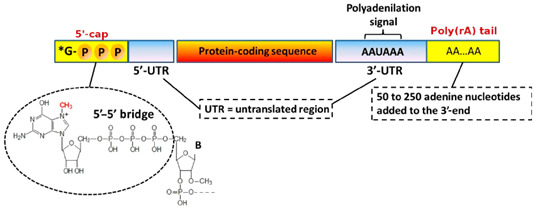	
**Element**	**Description**
Cap (2)	A modified 5′-cap1 structure (m7G+m3′-5′-ppp-5′-Am): GA
5′-UTR (52)	The 5′-untranslated region derived from human alpha-globin RNA with an optimized Kozak sequence. GAATAAACTAGTATTCTTCTGGTCCCCACAGACTCAGAGAGAACCCGCCACC
Signal peptide (48)	S glycoprotein signal peptide (extended leader sequence) guides translocation of the nascent polypeptide chain into the endoplasmic reticulum: ATGTTCGTGTTCCTGGTGCTGCTGCCTCTGGTGTCCAGCCAGTGTGTG
Coding region (n)	Codon-optimized sequence (ORF). Replace U with Ψ, but mRNA sequencing projections require replacement with T for projections.
3′-UTR (268)	The 3′ untranslated region comprises two sequence elements derived from the amino-terminal enhancer of split (AES) mRNA and the mitochondrial encoded 12S ribosomal RNA to confer RNA stability and high total protein expression: GCTAGCTGCCCCTTTCCCGTCCTGGGTACCCCGAGTCTCCCCCGACCTCGGGTCCCAGGTATGCTCCCACCTCCACCTGCCCCACTCACCACCTCTGCTAGTTCCAGACACCTCCCAAGCACGCAGCAATGCAGCTCAAAACGCTTAGCCTAGCCACACCCCCACGGGAAACAGCAGTGATTAACCTTTAGCAATAAACGAAAGTTTAACTAAGCTATACTAACCCCAGGGTTGGTCAATTTCGTGCCAGCCACACCCTGGAGCTAGC
poly(A) (110)	A 110-nucleotide poly(A)-tail consisting of a stretch of 30 adenosine residues, followed by a 10-nucleotide linker sequence and another 70 adenosine residues: AAAAAAAAAAAAAAAAAAAAAAAAAAAAAAGCATATGACTAAAAAAAAAAAAAAAAAAAAAAAAAAAAAAAAAAAAAAAAAAAAAAAAAAAAAAAAAAAAAAAAAAAAAA
